# Electric Current Application on Dental Implant Biofilms: Review

**DOI:** 10.3390/jfb15070197

**Published:** 2024-07-17

**Authors:** Flávio Rodrigues, Mariana Rodrigues da Silva, Filipe S. Silva, Sara Madeira, Óscar Carvalho

**Affiliations:** Center for Micro-Electro Mechanical Systems (CMEMS), University of Minho, Campus de Azurém, 4800-058 Guimarães, Portugal; gabriel_rodrigues97@hotmail.com (F.R.); m.silva@dem.uminho.pt (M.R.d.S.); saracsoaresm@hotmail.com (S.M.); oscar.carvalho@gmail.com (Ó.C.)

**Keywords:** dental implant, biofilms, peri-implantitis, electrical current

## Abstract

The prevalence of complications due to the presence of biofilms in dental implant surfaces and their relationship with peri-implant diseases, namely peri-implantitis, remain difficult problems to overcome. The information available about the application of electric current on dental implant biofilms; its parameters, namely current level, voltage and exposure time; and related effects are still not enough to understand which individual mechanisms are caused by this technique, culminating in the decrease or eradication of the biofilm. The purpose of this narrative review, based on a systematic search, is to understand the effect of electric current directly applied to biofilms present in dental implants and which parameters are used. For the systematic search, electronic databases including MEDLINE/PubMed, Scopus, and Web of Science, up to and including November 2023, were searched. Seven studies were included. A 12-item checklist was used to assess their methodological quality. All studies used direct/constant electric current; however, that use was not achieved by the same protocol/set-up. Parameters such as current, voltage, resistance, and actuation time were different in all studies. Monospecies and multi-species biofilm were used in the substrate made of titanium. The results indicate that the use of constant and alternating electric current directly applied to dental implant’s surfaces is a promising way to treat problems related to biofilms and peri-implant diseases. Future trials, namely in vivo tests, are necessary to reveal all the potential of this treatment.

## 1. Introduction

The use of dental implants to treat partially or completely edentulous patients has been increasing in the last few years [1,2,3]. Despite the high rates of long-term success and survival, patients with implants can develop infections and inflammations in the peri-implant region, leading to implant failure [4,5].

Peri-implant microbiota plays an important role in the development of peri-implant diseases, namely peri-implantitis [6]. Peri-implantitis is a common complication involving infection and inflammation of the tissue around a dental implant. It is characterized by the inflammation of the peri-implant connective tissue and a progressive loss of the supporting bone [7]. This inflammatory condition is triggered by bacteria capable of forming biofilms on the implant surface, leading to peri-implant pocket formation, soft tissue degradation, and bone resorption [8,9]. The main microorganisms responsible for peri-implantitis include *Porphyromonas gingivalis*, *Tannerella forsythia*, *Treponema denticola*, *Aggregatibacter actinomycetemcomitans*, *Fusobacterium nucleatum*, *Streptococcus species*, *Prevotella intermedia*, and *Parvimonas micra* [10]. According to the literature [5,11], peri-implantitis affects approximately 10–20% of dental implants and can lead to implant failure if left untreated. 

Managing the biofilm present on the implant surface should be the first focus point to deal with this disease. It will be important that the strategy used does not damage or alter the surface integrity of the implant [12,13]. Various protocols, both non-surgical and surgical, have been documented for the purpose of reducing bacterial attachment and eliminating biofilm. These protocols include mechanical, laser, chemical, and photodynamic treatments [14,15,16]. Originally used to treat teeth, these methods have recently been applied to dental implants, as it is believed that bacterial colonization and biofilm play similar roles in implant and dental surfaces [17,18,19]. 

Despite the ability of the treatments mentioned earlier to reduce biofilm formation or slow its development, there is still no consensus regarding their effectiveness. Therefore, defining treatments and their protocols for inhibiting or reducing biofilm at a level where they are not harmful, or even eradicating it, remains a challenge [13,18,20]. In addition to the challenge of preventing biofilm formation, another reason for unsatisfactory results is the difficulty in decontaminating the implant surface. This difficulty arises from its structure, limited access, improper working angles and distances of the implant, and the ability of microorganisms to form communities (biofilms), making them more resistant to antimicrobial agents and the host immune system [16,19]. 

Several novel treatment approaches have been developed to address these challenges. One innovative method, discussed in the literature, involves applying an electric potential or electrical current to prevent and/or treat biofilm [21,22]. This field of investigation has been in constant development. Some reports refer to the therapeutic use of an electric current capable of killing planktonic bacteria [23]. Two distinct approaches can be identified: the bioelectric effect and the electricidal effect. The bioelectric effect involves the combined use of an electric current or an electric field with antimicrobials to enhance their efficacy. Conversely, the electricidal effect refers to the use of electric current alone to kill bacteria [23,24]. The influence of bioelectric mechanisms on biofilms is wide-ranging, affecting their structure, viability, and resistance. Bioelectric therapies have been shown to disrupt the integrity of biofilm matrices, increase the permeability of bacterial cell membranes, and improve the efficacy of antimicrobial agents [25]. However, several critical issues remain unresolved, including determining the optimal electrical parameters [26]. Furthermore, there is a need to explore the specific interactions between electric fields and biofilm components, such as extracellular polymeric substances, as well as to understand how biofilm heterogeneity impacts treatment effectiveness. 

Therefore, a comprehensive review of the literature reports is necessary to understand the relationships between protocols, testing parameters, and outcomes associated with this treatment approach. Examining various parameters such as intensity, duration, and point of application of an electric current is essential for understanding its effects on bacterial prevention and biofilm eradication. Hence, this review aims to explore how applying an electrical current to dental implant surfaces can prevent biofilm formation. It will also examine the various parameters and protocols used in this approach.

## 2. Materials and Methods

A review of the literature using a systematic search was carried out to assess the outcomes on bacteria/biofilm from applying electric current in dental implant surfaces and what parameters are used in that kind of strategy. 

### 2.1. Search Strategy

Electronic databases including MEDLINE/PubMed, Scopus, and Web of Science were searched up to 26 November 2023, to identify articles that included the following terms: “dental implant”, antifouling, bacterial, biofilm, “peri-implantitis”, current, electr*, potential, voltage, biocidal, clean, decontamination, decrease, detachment, desorption, elimination, eradication, killing, inactivation, inhibition, mitigation, prevention, removal, stimulation, treatment, and therapy. An example of the search strategy in PubMed database is presented in “Appendix A”. 

### 2.2. Study Selection (Inclusion and Exclusion Criteria)

All records were extracted to an Excel file (Microsoft^®^ Office Version: 18.2407.1052.0) and Mendeley Desktop (version 1.19.4) and duplicates were removed by software filter and then verified manually. The references of relevant studies were searched to look for other studies as a secondary search, which could potentially be relevant to this review. At this point, 5 more studies were added to the search. Titles, abstracts, and keywords were screened, and relevant studies were retrieved for full-text analysis. Studies analyzing the effect of electric potential/current on dental implants and studies that worked with biofilms and electric current, even if it was not in the form of dental implants, were considered for inclusion. Studies were excluded if they did not (1) have any relation to electric potential/current directly applied into dental implant or implant surface, (2) have any relation to any type of electric potential/current, or (3) have any relation to biofilm treatment/eradication/reduction. Studies that were not written in the English language or where a full-text version was not found were also excluded. 

### 2.3. Data Collection and Extraction 

To extract key details from articles included in the qualitative synthesis, data extraction tables, in which each article was identified by first author and year, were developed considering the inputs and the outputs of the study. For the inputs, the following parameters were considered: (1) objective; (2) treatment type; (3) biofilm substrate (local to bacteria growth) and dimensions; (4) electrodes, electrolyte, or medium; (5) microorganisms; (6) current, voltage, resistance, and exposure time. Regarding the outputs, (1) laboratory analysis (protocol to analyze the effect of electric current) and (2) the effect that electric current produced on the biofilm were considered. These points were chosen to facilitate the comparison between methodologies and parameters used in each study, as well as the results and conclusions obtained. 

### 2.4. Risk of Bias Assessment

The risk of bias in the included studies was evaluated using the Risk of Bias Assessment tool for Non-randomized Studies (RoBANS). RoBANS is a validated instrument designed to assess the risk of bias in non-randomized in vitro studies. It comprises seven domains: “bias due to confounding”, “bias in selection of participants into the study”, “bias in classification of interventions”, “bias due to deviations from intended interventions”, “bias due to missing data”, “bias in measurement of outcomes”, and “bias in selection of the reported result”. The criteria for each domain were tailored to fit the context of our systematic review, particularly to evaluate the risk of bias in in vitro studies [27]. Table 1 describes the criteria used to judge the risk of bias of each domain. Two authors (FR and SM) independently evaluated and classified the risk of bias in all included studies. 

## 3. Results

### 3.1. Search Strategy—Results

**Figure 1:**
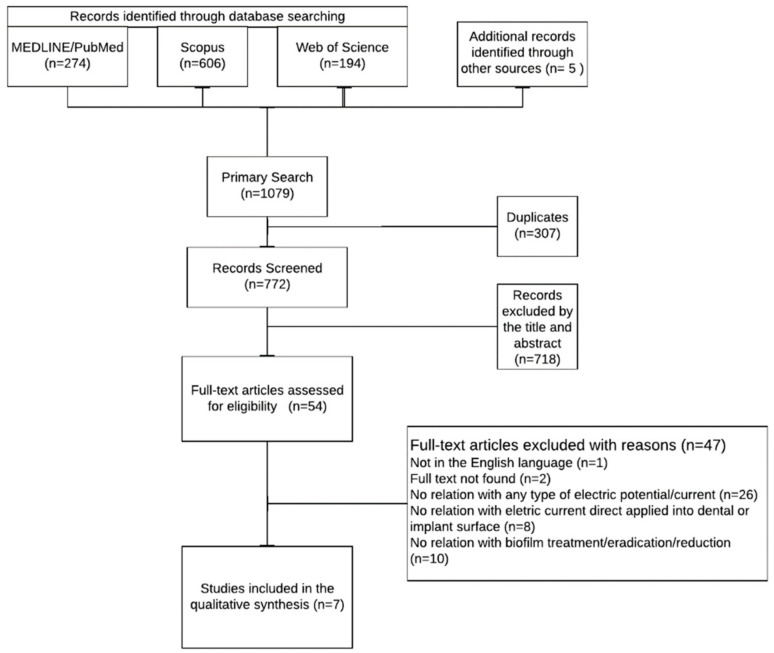
Flowchart of the present search strategy.

After a primary search, 1079 articles were found, and 307 duplicates were removed. Titles, abstracts, and keywords were screened, and 718 articles were excluded. For full-text analysis, 54 relevant studies were retrieved. Seven studies were included for the qualitative synthesis. The study selection process is summarized in Figure 1.

### 3.2. Risk of Bias 

**Figure 2:**
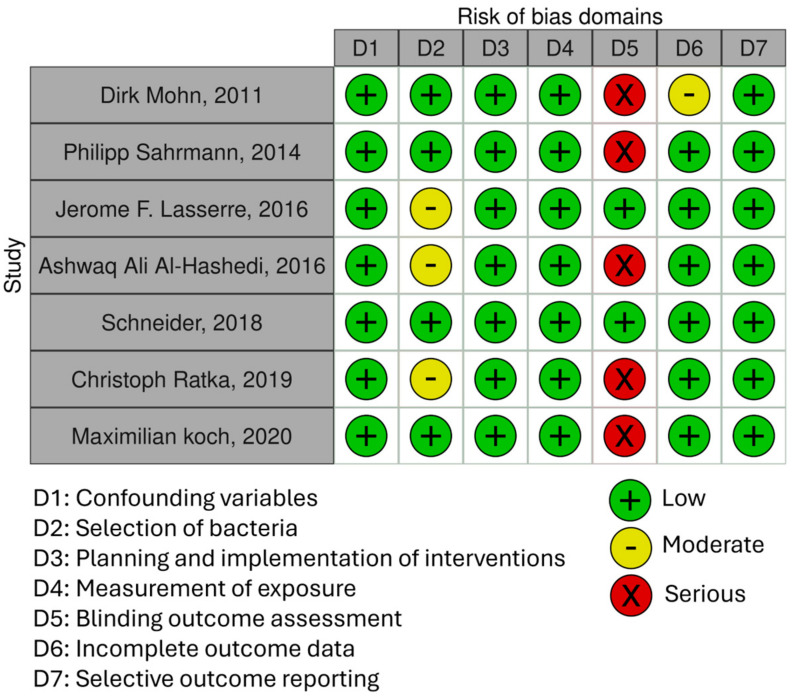
Risk of bias plots. Traffic lights plots for in vitro studies: Dirk Mohn, 2011 [17], Philipp Sahrmann, 2014 [21], Jerome F. Lasserre, 2016 [18], Ashwaq Ali Al-Hashedi, 2016 [13], Schneider, 2018 [30], Christoph Ratka, 2019 [28] and Maximilian Koch, 2020 [29].

The judgment of the risk of bias for each in vitro study and a summary for each domain is displayed in Figure 2. The “Confounding Variables” domain was judged as having a low risk of bias for all in vitro studies. The “Selection of Bacteria” domain presented a moderate risk of bias in three in vitro studies [13,18,28] that did not report the bacteria used. The “Planning and Implementation of Interventions” and “Exposure of Measurement” domains were judged as having a low risk of bias. The “Blinding Outcome Assessment” domain was judged as presenting a serious risk of bias in five studies [13,17,21,28,29] due to an inadequate measurement of semiquantitative or qualitative outcomes. The “Incomplete Outcome Data” and “Selective outcome data” domains were judged as low risk for all in vitro studies, except for one study ([17]) that is of moderate risk because the sample size is not clear.

### 3.3. Type of Treatment and Objective

The authors of the selected in vitro studies named the treatments differently (Electrolysis [17,21,30], DC application [18], Electrolytic [28], Electrochemical [13,29]), although similarities could be found between them. Despite all the studies using electric current and evaluating its impact on biofilms, for some studies this was not the main objective. Thus, the studies can be divided into three groups. First, a group with studies [13,17,21,30], in which the main objective was to evaluate what occurs in the biofilm, previously formed in the substrate to be used, with the application of electric current. In this group, some samples are test samples (samples with treatment) while others are control samples (samples without treatment), and in the end, they are compared (test and control) for the results. A second group, in which the use of electric current as a treatment for biofilm, previously formed, is compared to other forms of treatment in order to realize which of these obtains better results, includes studies [28,29]. There is a comparison between forms of treatment, instead of a comparison between samples with treatment and without treatment, as occurs in the first group. And finally, the remaining study [18], in which the main objective is to evaluate the effect of electric current when used simultaneously with chlorhexidine (CHX). However, in that study, a trial was carried out in which the use of electric current is evaluated alone, and because of that, the study can be included in the present review. 

### 3.4. Substrate, Electrodes, and Microorganisms

**Figure 3:**
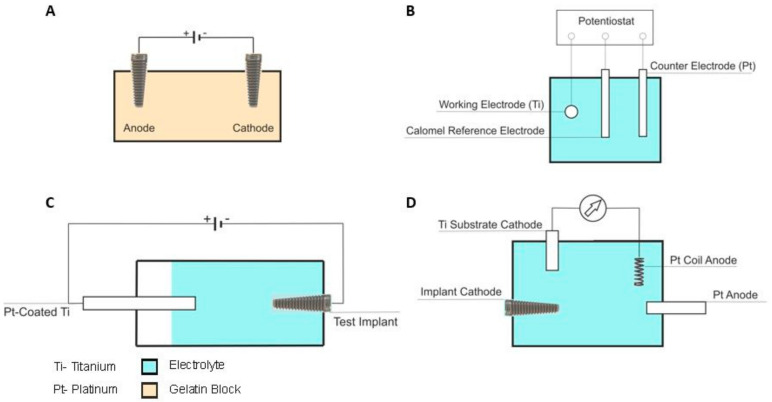
Schematic representation of some experimental set-ups adapted from the following: (**A**)—Mohn, D. et al. [17]; (**B**)—Al-Hashedi, A. et al. [15]; (**C**) Ratka, C. et al. [28]; (**D**)—Schneider, S. et al [30].

All studies, with the exception of study [29], used titanium as a substrate (for the biofilm) and electrode. They were the same, though some were in implant form or the form of representative implant discs. Even in studies [13,30], where a three-electrode electrochemical cell was used, the working electrode was titanium. In studies [21,29,30], the substrates were the implants commercially available. The solutions of the medium in which the substrates were found were different in all selected studies, as can be seen in Table 2. Regarding the experimental set-ups, it was found that these were different between studies. In Figure 3, it is possible to see a schematic drawing of some experimental set-ups used. Studies [17,21,29,30] used defined species of bacteria to produce biofilm (Table 2), while studies [13,18,28] used human saliva for the formation of biofilm. In these last three studies, the present species were not specified.

### 3.5. Electric Current Parameters (Current, Voltage, Resistance, and Exposure Time)

Regarding the electric current used, the studies can be divided into two groups: one group where the current value used for each test was constant, although the current value could vary between tests, and another group [28,29] where the current values were variable for the same test. All selected studies used an external source (Table 1) for the electric current supply, except for study [29] where nothing is mentioned about this topic.

In the work conducted by Dirk Mohn et al. [17], current values of 2, 5, 7.5, and 10 mA were used. P. Sahrmann et al. [21] used a current value of 10 mA for all tests. In these two studies [17,21], it was mentioned that the power source varied the voltage so that the current remained constant. For these studies, the voltage ranges were 4–20 V and 11–19 V, respectively. The resistance for both studies ranges from 2 to 6 Ω. Jérôme Lasserre et al. [18] mentioned that their source only allowed the application of a current equal to 5 mA. In work conducted by S. Schneider et al. [30], they mentioned a constant application of current (300 mA), despite the possibility to change the voltage (although they presented a value of 7 V for this parameter), and there was no reference about how they kept the current constant. C. Ratka et al. [28] and M. Koch et al. [29] applied a current range up to 1100 mA and 5–22 mA, respectively, and a voltage value of 6 V common to both. Finally, in the remaining study, Al-Hashedi et al. [13] used a constant current and voltage; however, that study differs from the others because the current applied to the anode and cathode was of different values. A current of 2.3 mA was applied to the cathode and 22.5 µA to the anode with a potential of ±1.8 V. Thus, the values ranged from 22 µA to 1100 mA for current, from 1.8 to 20 V for voltage, and from 2 to 6 ohm for resistance. Regarding exposure time, studies [13,17,18,21,28,30] used a certain time that was kept constant for each test, while study [29] referred to the use of a variable time between 5 and 60 min (Table 1).

### 3.6. Protocol to Access the Bacteria Viability

After the experiments, to measure the results, the laboratory analysis was conducted to realize how much biofilm was kept alive. In this way, study [17] compared the number of viable cells in the tested samples with the control samples. In [21], confocal laser scanning microscopy was used to verify the results of a live/dead BacLight bacterial viability assay. In [18], the authors used a computer-assisted device (Acolyte, from Synbiosis^®^, Frederick, MD, USA) to automatically count the colonies, and in studies [13,28], SEM was used to check the number of CFUs (colony-forming units). Study [30] used a live/dead assay, while in [29], the bacterial growths on infected tested implants with different treatments were compared.

### 3.7. Obtained Results

The results obtained were always compared with the control groups. D. Mohn et al. [17] and P. Sahrmann et al. [21] made a distinction between the results obtained in the anode and the cathode. In both studies, complete disinfection was obtained for the anode. On the other hand, in the cathode, a reduction of 99% in total counts, i.e., an almost complete disinfection, was obtained in [17]. In [21], there was a reduction of 28.5 to 71.4% in total counts. Other studies [13,30] also obtained complete disinfection of the samples. However, it should be noted that in [13], after the application of electric current, a mechanical Ti disinfection brush was used. These sequential and combined strategies allowed for a complete disinfection. For the work by Jérôme Lasserre et al. [18], the disinfection obtained was about 58.5%. In the study by C. Ratka et al. [28], colony-forming units (CFUs) were not found in the test samples, contrary to what occurred in the control group, which indicates that there was no development of biofilm. In that study [24], the main objective was to compare the cleaning effect of two different treatments on titanium implants. Thus, the electrolytic cleaning, i.e., using electric current, was the test group method and would be compared with the control group (power spray system cleaning). M. Koch et al. [29] tested three different cleaning techniques (mechanical debridement, air abrasion, and electrochemical disinfection). The results showed that electrochemical disinfection is more effective for the species *C. dubliniensis* when compared to the other methods if the treatment times of boron-doped diamond (BDD) electrodes were increased. For *E. faecalis*, BDD electrodes showed complete disinfection, unlike in the other treatments. Finally, for multi-species biofilm, electrochemical disinfection did not achieve complete disinfection, but, again, it showed better results than the other two treatments to which it was the subject of comparison.

## 4. Discussion

This narrative review focuses on two main issues: (1) What is the effect of electric current on biofilms formed in dental implants? and (2) What are the main parameters of electric current used for the treatment of biofilms? This subject has been studied mainly through in vitro experiments, using biofilms formed in the laboratory and whose composition presents one or more species of bacteria. Overall, the studies have shown a significant reduction of biofilm when only electric current is used or when it was applied simultaneously with other treatments such as, for example, the use of antibiotics [18]. The most important aspects to retain are the parameters used for the treatment with electric current, namely, the current (amperes), its exposure time, and its mode of action, which is the way the electric current acts in biofilm to decrease its prevalence. 

The interest in electrical stimulation and its effects on humans has been increasing due to a broader knowledge of the properties of tissues and cells. This type of stimulation has been used in various fields of medicine. For example, to treat brain damage, the nervous system, and cardiology, among many others [31]. It has also been demonstrated that direct current has some efficacy in killing planktonic bacteria in static and flowing systems [32]. Much has been hypothesized regarding the mechanism of current action in bacteria. It is known that bacteria are sensitive to the passage of electrical current because this induces a transient permeability of the cell membrane; however, the mechanism that leads to bacteria death is not yet fully understood [31,33,34]. 

Direct and indirect consequences of electric current have been indicated as possible mechanisms [31,35]. Direct actions indicate that the death of bacteria occurs due to the damage that electric current causes to the cell membrane, affecting its permeability or even blocking the multiplication of bacteria cells if the current is used simultaneously with anti-microbials. The combined action of anti-microbials and current also leads to the death of the bacteria [31,33,35]. The consequences of the application of electric current, some indirect, are also considered as possible mechanisms that lead to bacteria death. Among these are the products of electrolysis, galvanotaxis (which refers to the increased migration of white blood cells such as leukocytes and macrophages, belonging to the immune system, which will act against the bacteria), change of pH, and temperature. However, temperature change, with the application of electric current, has already been demonstrated [36] to not influence the death of bacteria because its variation is not significant enough [31]. 

The pH change resulting from electrolysis products (production of toxic substances such as H2O2, oxidizing radicals, and chlorine molecules, among others) seems to be a decisive factor in the death of bacteria. This varies between alkaline at the cathode and acidic at the anode. This difference can be explained by the electrolysis products that differ between the anode and the cathode. Moreover, in the cathode, with the formation of gas, and in the anode due to corrosion and discoloration, the detachment and inhibition of the growth of bacteria could be facilitated [35]. In addition, the study by D. Mohn et al. [17] stated that the higher the electrical charge, the greater the killing efficiency; however, it is always necessary to be careful with what the human body can support. All this supports what is referred to in the selected studies. 

**Figure 4:**
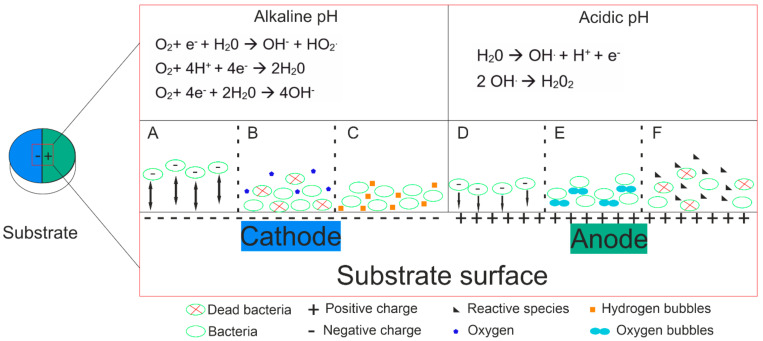
Some proposed biofilm-associated reduction mechanisms: (**A**) electro-repulsive forces; (**B**) low oxygen due to reactions lead to aerobic bacteria death; (**C**) hydrogen bubbles lead to bacteria detachment; (**D**) electrostatic forces; (**E**) oxygen bubbles lead to bacteria detachment and (**F**) interaction between Reactive Oxygen Species and bacteria lead to cell death.

Overall, the presented mechanisms responsible for biofilm reduction are schematically represented in Figure 4.

To emphasize that, in the anode, due to the formation of active oxidants and the evolution of oxidative species, there was a greater decrease in the number of bacteria when compared to the cathode, where the pH is more alkaline and can be partially supported by some bacteria [17]. 

On the other side, cathodic potentials can produce electro-repulsive forces, causing the detachment of the biofilm in the opposite of what occurs with anodic potentials. In fact, according to Poortinga [37], bacterial adhesion is achieved essentially due to three main forces: electrostatic, electrophoretic, and electroosmotic. If repulsive forces (that are opposed to adhesion forces) between the negative surface charge of bacteria and the cathodic surface were applied, detachment of the biofilm will occur [38]. 

It was previously mentioned that anodic (negative potential) and cathodic (positive potential) currents contribute in different ways to the reduction of biofilm. Therefore, a solution for better use of the electric current for the treatment of biofilm may be the use of both potentials. In this way, it will be possible to combine the greater anodic current inactivation effect with the greater cathode current detachment effect. However, it is necessary that when the bacteria are inactivated and/or detached, they are removed from the site because they become floaters. At this point, the body’s defenses and antibiotic therapy can easily destroy the bacteria, but it can also be used with complementary methods such as an antiseptic solution and mechanical toothbrush. If this does not happen, the detached bacteria can accumulate again on the surface by deposition or even serve as seeds for the adhesion of new bacteria [38].

The use of electric current to treat problems like biofilms seems to have a promising future. However, to carry out these in vivo studies, it must be considered that the current to be used cannot cause damage to the human body and the animals involved in these types of studies. However, this current limit is not established. German standardization [39] states that small levels of current can trigger a sensitive perception by the human body when a part of it touches or releases a current source. This type of sensory perception can still be felt during the passage of current. For example, a slight tingling is felt at 1.1 mA, and between 6 and 16 mA there occurs a painful shock and the loss of muscular control. Fish et al. [40] and Raikar et al. [41] suggested the limit of 10.5 mA of AC or 88 mA of DC [13] as the hazardous current limit in their studies. According to this, only two studies in the present review [28,30] do not respect this current limit tolerable by the human body. Friederike Kaiser et al. [42] studied the success and side effects of different treatment options in the low current attack of bacterial biofilms on titanium implants and used current densities in the range of 0.25 mA/cm^2^ to 2 mA/cm^2^. For an implant with an approximate area of 1.8 cm^2^, this means an application of a current between 0.45 and 3.6 mA. Friederike Kaiser et al. [42] stated that at 0.5 mA/cm^2^ approximately 98% of all bacteria were killed.

Another issue to be discussed is related to current density. The current density is dependent on the area covered. Accordingly, the shape of the object through which the electric current will pass has some impact on the amount of current that flows to a given point. 

Following this line of thought, that the current is area-dependent, in the selected articles, either to allow a better comparison with other studies or for a better explanation/measurement of the results, the authors should work with current density instead of Amperes, Volts, and Ohms. That is because, in the selected studies, the authors made use of some of the previously mentioned measurement units; however, they did not provide other values such as the area that is covered or the resistance. Without this complementary information, current density could not be calculated and the comparison between studies is hampered.

In addition to the previously raised points, other parameters such as the distance between electrodes and the number of electrodes is not specified in the selected articles. The distance is important because the passage of current is also dependent on the characteristics of the materials it flows. Thus, there are always associated energy losses. Consequently, the current released by the source will not be the same as that which reaches a point close to it or a point distant from it.

There are some limitations associated with the use of electric current for this type of application. One of them, as already mentioned, is related to the fact that the currents used can stimulate nerves and muscles leading to pain, discomfort, and unwanted muscle contractions, which can be overcome using local anesthesia in clinical use. Another limitation is that, if the location that needs treatment is deep, unless there is an electrical conductor to that location, the current density needed for that location to deliver the proper current will have to be higher. Hence, a higher current density than required can cause damage. Also, direct current (DC) cannot be generated by isolated electrodes [43], so it is always necessary for wires to connect to the source.

Several currents with different exposure times were used and showed good results, as in the study by Mohn et al. [17]. However, it is necessary to consider that the studies were not uniform. There are several different variables among them (different currents, different set-ups, different biofilms) which do not allow for a more faithful data comparison. Making use of higher current, when it can be applied, will bring advantages, since the higher the current, the higher the efficiency [35]. Among the values reported regarding the current and their respective results/effects, 5 mA is within the range of values observed in this review and seems to be promising for conducting in vitro tests. For the level of exposure time, it was verified that sometimes the complete disinfection of the biofilm was achieved before the stipulated time for the test. Therefore, using the treatment for a longer period may be unnecessary as the disinfection can be achieved earlier. However, to ensure that disinfection can indeed be carried out, the use of a longer exposure time makes perfect sense. Therefore, an exposure time of 10 min is also within the range of values observed in this review and it may be promising for conducting in vitro tests. Regarding biofilm, the use of saliva to promote biofilm formation is probably the most suitable choice. Thus, it will be possible to simulate human oral conditions. However, using this option, it would be important to try to understand which species are present in this biofilm formed from saliva to obtain more concrete results. To finish, it would be interesting too if the set-up for this kind of study was always the same, to allow a better comparison between different studies, decreasing, in this way, the dependent variables.

The application of electric current on dental implants for treating biofilms presents both advantages and disadvantages. Electric current can effectively disrupt biofilms and inhibit bacterial growth, offering a promising approach to prevent and treat peri-implant infections. The non-invasive and localized nature of the treatment make it an attractive option for patients, especially those who may not tolerate invasive procedures such as mechanical debridement, which is one of the most used techniques. However, challenges such as limited penetration into deeper biofilms, potential tissue damage (if not properly controlled), and variable efficacy need to be addressed. The clinical application of electric current involves techniques like direct current application, pulsed electric fields, and electrochemical treatment, often combined with antimicrobial agents for enhanced effectiveness. Future research should optimize treatment protocols in terms of the length of time of application, current intensities, and the strategies to apply electric current in a customized way according to different patient requirements. Extensive clinical trials and mechanistic studies are needed to evaluate long-term outcomes and refine approaches. Establishing regulatory guidelines and training programs could ensure safe and effective clinical use, potentially standardizing electric current therapy for managing peri-implant infections.

## 5. Conclusions

Dental implants can be invaded by bacteria, causing the subsequent development of biofilms that can lead to peri-implant mucositis and then, eventually, to peri-implantitis.

The use of electric current, although the parameters were diversified, has demonstrated a significant reduction of biofilms in dental implants’ surface specimens.

The use of anodic and cathodic currents together seems to be more efficient.

Parameters such as 5 mA for the electric current and 10 min for the exposure time stand out and could be suitable for a successful treatment. 

Although this type of approach is promising, further studies, namely in vivo studies, should be conducted to attest its applicability, at a more advanced stage, in humans. In this sense, the results obtained can be used as a starting point.

## Figures and Tables

**Table 1:** Domains and description for the appraisal of the risk of bias for in vitro studies using Risk of Bias Assessment tool for Non-randomized Studies (RoBANS).

Domain	Description for In Vitro
Confounding variables	Selection bias caused by inadequate confirmation and consideration of confounding variables. For in vitro studies, bacteria should be used from the same strain and the same growth protocol for all experimental groups. Same experimental conditions should be guaranteed for both control and intervention groups (temperature conditions).
Selection of Bacteria	Selection bias caused by inadequate confirmation and consideration of confounding variables. For in vitro studies, selection of bacteria should be performed from commercially available strains or from biofilm samples collected from humans. In the second case, the biofilm composition should be clearly described.
Planning and Implementation of Interventions	Performance bias caused by inadequate planning and implementation of interventions. Techniques used to study the electric current effects should be adequate and well-established for the specific outcomes that studies are assessing, and their measurement protocol should be clearly described to allow for replication. Semiquantitative and/or qualitative analysis should be performed by two independent observers to ascertain interoperator reliability.
Measurements of Exposure	Performance bias caused by inadequate measurement of exposure. Measurement techniques should be adequate and well-established for the specific outcomes that studies are assessing, and their measurement protocol should be clearly described to allow for replication. Semiquantitative and/or qualitative analysis should be performed by two independent observers to ascertain interoperator reliability.
Binding Outcomes Assessment	Detection bias caused by inadequate blinding of outcome assessment. Outcome assessor and/or data analysist not blinded to group (i.e., intervention vs. control). For quantitative analyses, the blinding of outcome assessor and/or data analyst was not considered necessary. Otherwise (semiquantitative and qualitative analyses), blinding was required.
Incomplete Outcome Data	Attrition bias caused by inadequate handling of incomplete data outcome.Missing data from what is proposed in methodological section.
Selective Outcome Data	Reporting bias caused by selective outcome reporting. Evaluate whether the reported results might be selective, focusing only on positive findings while omitting negative or null results.

**Table 2:** Overview of the data extraction criteria for each study included in the present review.

Article		Inputs					Outputs		
First Author (Year)	Hypothesis/Objectives	Treatment Type	Substrate	A-Electrodes B-Electrolyte or Medium	Microorganism	A-Current (mA) B-Voltage (V) C-Resistance D-Exposure Time	Lab Analysis	Effect	
								Anode	Cathode
Dirk Mohn (2011) [17]	Electrolysis can reduce viable counts of adhering bacteria and this reduction should be greater if active oxidative species are generated	Electrolysis	Standard Dental Titanium Implants (Straumann SLA, Straumann AG, Basel, Switzerland) with 4.1 diameter and 12.0 in length	A-Titanium implants B-Physiological saline	*E. coli*	A-Continuous (2; 5; 7.5; 10 mA) B-4–20 V C-2–6 KΩ D-15 min each current	Colony-forming units count of viable bacteria compared to positive control treatments	Complete kill of CFUs	99% kill of CFUs
Philipp Sahrmann (2014) [21]	A low, direct current should suffice to eradicate viable counts on implant surfaces, and that electrolytic disinfection should be more thorough on anode implants than on cathode counterparts	Electrolysis	Titanium discs with a sandblasted, acid-etched, large-grit titanium surface (SLA; Straumann, Basel, Switzerland) with an overall surface of 4.0 cm^2^	A-Titanium discs B-0.9% NaCL solution as conductive liquid	*Streptococcus oralis*; *Streptococcus anginosus*; *Actinomyces oris*; *Fusobacterium nucleatum*; *Veillonella dispar*; *Campylobacter rectus*; *Prevotella intermedia*; *Porphyromonas gingivalis*	A-Constant of 10 mA B-11–19 V C-2–6 KΩ D-10 min each disc	Colony-forming units count (confocal laser scanning microscopy was used with live/dead BacLight bacterial viability assay)	Complete kill of CFUs	28.6–71.4% kill of CFUs
Jérôme F. Lasserre (2016) [18]	To test ex-vivo the influence of 5 mA direct electric current on the antimicrobial efficacy of CHX against human dental biofilms grown in vivo on titanium or HA surfaces.	DC application	Grade 5 (TiAl6V4) machined Ti discs with 5.0 diameter and 2.0 width	A-Titanium discs B-Phosphate Buffered Saline	-Biofilm formed in vivo (five healthy volunteers)	A-5 mA DC B-Not given C-Not given D-5 min each disc	Colony-forming units count (computer-assisted device)	At 5 min, the proportion of killed bacteria compared with baseline was more than twice as in the control group with a percentage of viability reduction increasing up to 58.5%	
Ashwaq Ali Al-Hashedi (2016) [13]	To investigate if electrochemical treatments with alternating potential are able to both remove organic contamination and bacteria from Ti implant surfaces	Electrochemical	Titanium discs with 10 mm diameter and 1 mm thickness	A-A three-electrode electrochemical cell was set up as follows: a saturated Hg/HgCl calomel reference (SCE), a platinum wire counter, and a Ti disc working electrode B-All electrodes were immersed in an electrolytic solution and the electrochemical measurements were performed using a potentiostat	-Oral biofilm formed in six humans and saliva	A-Cathode 2.3 mA anode 22.5 uA B-1.8 V C-Not given D-5 min (2.5 anodic 2.5 cathodic)	Colony-forming units count (scanning electron microscopy images)	Complete removal of thick biofilms required adjunctive mechanical cleaning using Ti brushes	
Schneider (2018) [30]	Beneficial effect of electrochemical removal of *E. coli* biofilms by the hydrogen evolution reaction (HER) at titanium surfaces in combination with the in-situ generation of a disinfecting agent.	Electrolysis	Titanium substrate with 10.0 length and 10.0 width	A-Titanium dental implant (Straumann BL Ø 4.1 mm, RC SLA™, Grade 4, L: 11 mm), custom-built titanium disc electrodes (Ø 3 mm), or freshly prepared titanium substrates as cathode and a platinized titanium rod (Custom-built, Ø 4.0 mm, L: 10 mm) and Pt coils as anode B-Many types	*-E. coli K12 (JM101)* *-E. coli-GFP (HB101)*	A-Constant of 300 mA B-7 V C-Not given D-30 s	Colony-forming units count (LIVE/DEAD™ assay)	Complete disinfection	
Christoph Ratka (2019) [28]	To investigate the cleaning effect of an electrolytic approach (EC) compared to a powder-spray system (PSS) on titanium surfaces	Electrolytic	Grade 4 and 5 titanium design (like a standard parallel-threaded dental implant) with the following measures: (∅ 4.0 mm/L 11.0 mm pitch = 0.6 mm)	A-Titanium implants B-(sodium iodide (200 g/L), potassium iodide (200 g/L), L(+)-lactic acid (20 g/L), and water (800 g/L))	-Saliva (no specific microorganism)	A-Up to 1100 m B-6 V C-Not given D-5 min	Colony-forming units count	It was not possible to breed bacteria after the implants had been cleaned by the electrolytic approach = completely disinfection	
Maximilian Koch (2020) [29]	Comparison of 3 different types of treatment (air abrasion, mechanical debridement, and boron-doped diamond (BDD) electrodes	Electrochemical	Dental implants (straumann bone level taperes) with 4.1 × 12 mm	A-Diamond coating with boron doping of thin niobium wires (200 µm in diameter) B-Phosphate Buffered Salline	*-Bacillus pumilus*; *Bacillus subtilis*; *Enterococcus faecalis*; *Roseomonas mucosa*; *Staphylococcus epidermidis*; *Streptococcus sanguinis*; *Candida albicans*; *Candida dubliniensis*	A-5–22 mA B-6 V C-Not given D-5 to 60 min (variable)	Comparison of growth on Columbia Blood Agar plates after different treatment of implants infected	For *C. dubliniensis* and multi-species biofilm, electrochemical disinfection shows better results than the other methods; for *E. faecalis*, BDD shows complete disinfection	

## Appendix A

**Table A1:** Example search strategy for the PubMed database. The asterisk (*) is used as a truncation symbol in search strategies. It allows for the inclusion of multiple word variations that start with the same root. For example, using “electr*” in a search query will capture terms like “electric”, “electrical”, “electricity”, “electron”, among others.

Search	Search Terms	Results
#1	“dental implant”	8777
#2	antifouling	4.07
#3	bacterial	1,199,480
#4	biofilm	54,373
#5	“peri-implantitis”	2659
#6	#2 OR #3 OR #4 OR #5	1,226,588
#7	current	1,718,619
#8	electr *	4,341,572
#9	potential	3,019,019
#10	voltage	141,184
#11	#7 OR #8 OR #9 OR #10	7,695,330
#12	biocidal	75,983
#13	clean	74,683
#14	decontamination	14,498
#15	decrease	2,488,989
#16	detachment	64,329
#17	desorption	45,663
#18	elimination	330,003
#19	eradication	65,981
#20	killing	149,656
#21	inactivation	221,133
#22	inhibition	1,662,602
#23	mitigation	83,276
#24	prevention	2,574,281
#25	removal	638,425
#26	stimulation	1,326,089
#27	treatment	11,304,504
#28	therapy	9,692,170
#29	#12 OR #13 OR #14 OR #15 OR #16 OR #17 OR #18 OR #19 OR #20 OR #21 OR #22 OR #23 OR #24 OR #25 OR #26 OR #27 OR #28	15,128,975
#30	#1 AND #6 AND #11 AND #29	281
